# Biomarkers of potential harm in people switching from smoking tobacco to exclusive e‐cigarette use, dual use or abstinence: secondary analysis of Cochrane systematic review of trials of e‐cigarettes for smoking cessation

**DOI:** 10.1111/add.16063

**Published:** 2022-10-21

**Authors:** Jamie Hartmann‐Boyce, Ailsa R. Butler, Annika Theodoulou, Igho J. Onakpoya, Peter Hajek, Chris Bullen, Nancy A. Rigotti, Nicola Lindson

**Affiliations:** ^1^ Nuffield Department of Primary Care Health Sciences University of Oxford Oxford UK; ^2^ Department for Continuing Education University of Oxford Oxford UK; ^3^ Wolfson Institute of Population Health, Barts & The London School of Medicine and Dentistry Queen Mary University of London London UK; ^4^ National Institute for Health Innovation University of Auckland Auckland New Zealand; ^5^ Tobacco Research and Treatment Center, Department of Medicine Massachusetts General Hospital and Harvard Medical School Boston Massachusetts USA

**Keywords:** Electronic cigarettes, biomarkers, carbon monoxide, tobacco, smoking cessation, systematic review

## Abstract

**Aims:**

This study aims to compare biomarkers of potential harm between people switching from smoking combustible cigarettes (CC) completely to electronic cigarettes (EC), continuing to smoke CC, using both EC and CC (dual users) and using neither (abstainers), based on behaviour during EC intervention studies.

**Design:**

Secondary analysis following systematic review, incorporating inverse variance random‐effects meta‐analysis and effect direction plots.

**Setting:**

This study was conducted in Greece, Italy, Poland, the United Kingdom and the United States.

**Participants:**

A total of 1299 adults smoking CC (nine studies) and provided EC.

**Measurements:**

Measurements were conducted using carbon monoxide (CO) and 26 other biomarkers.

**Findings:**

In pooled analyses, exhaled CO (eCO) was lower in EC versus EC + CC [mean difference (MD) = −4.40 parts per million (p.p.m.), 95% confidence interval (CI) = −12.04 to 3.24, two studies] and CC (MD = −9.57 p.p.m., 95% CI = −17.30 to −1.83, three studies). eCO was lower in dual users versus CC only (MD = −1.91 p.p.m., 95% CI = −3.38 to −0.45, two studies). Magnitude rather than direction of effect drove substantial statistical heterogeneity. Effect direction plots were used for other biomarkers. Comparing EC with CC, 12 of 13 biomarkers were significantly lower in EC users, with no difference for the 13th. Comparing EC with dual users, 12 of the 25 biomarkers were lower for EC, and five were lower for dual use. For the remaining eight measures, single studies did not detect statistically significant differences, or the multiple studies contributing to the outcome had inconsistent results. Only one study provided data comparing dual use with CC; of the 13 biomarkers measured, 12 were significantly lower in the dual use group, with no statistically significant difference detected for the 13th. Only one study provided data on abstainers.

**Conclusions:**

Switching from smoking to vaping or dual use appears to reduce levels of biomarkers of potential harm significantly.

## INTRODUCTION

Scientific consensus overwhelmingly holds that nicotine electronic cigarettes (ECs), although not risk‐free, are considerably lower risk than combustible tobacco, as they provide a means to inhale nicotine without combustion [1, 2]. However, uncertainty persists around the health impacts in people who smoke who switch to EC completely, or who both smoke and use ECs (dual users) [1]. Without long‐term safety data, biomarkers of potential harm are often used to inform best estimates about relative benefits and harms of ECs compared to combustible tobacco. Biomarkers of potential harm include exhaled carbon monoxide (eCO), known carcinogens and other toxicants identified by regulatory bodies [3].

Our Cochrane living systematic review of ECs for smoking cessation evaluates changes in biomarkers between groups randomized using an intention‐to‐treat principle [4]. This means that we compare differences in biomarkers of harm between those randomized to an EC condition and those randomized to a control condition. This follows standard Cochrane methods and is a pragmatic approach which best reflects the effects of such interventions delivered in real life. However, as with all smoking cessation trials, most people in these studies do not quit smoking successfully. In the EC condition, some participants will not use EC, and many will continue to use combustible cigarettes. In the control condition, some participants will use EC of their own accord. Most will also continue to use combustible cigarettes [4].

Although it is important to analyse the impact of the randomized interventions by group, there is also value in knowing how biomarkers of harm change based on participants’ actual behaviour. Such analyses can inform debates concerning potential harms and benefits of dual use. They can also illustrate the changes that someone who smokes might expect to see if they switched completely to ECs or commenced dual use. Therefore, in this secondary analysis of trials included in our Cochrane review, we set out to compare biomarkers of potential harm between four groups, based on actual product use rather than group assignment: those switching completely to ECs, those not using EC and continuing to smoke combustible cigarettes (CC), those using both EC and CC (dual use) and those using neither (abstainers).

## METHODS

This is a secondary analysis using data identified to January 2022 in a Cochrane living systematic review [4]. The following databases were searched: Cochrane Tobacco Addiction Group Specialized Register, Cochrane Central Register of Controlled Trials, MEDLINE, Embase, PsycINFO, ClinicalTrials.gov and WHO International Clinical Trials Registry Platform. Full search methods can be found in the parent review [4]. A protocol was registered in advance (https://osf.io/g6bw3/).

### Inclusion criteria

Inclusion criteria were as per the parent review; namely, studies of EC interventions provided to adults who smoke for the purpose of smoking cessation, including randomized controlled trials, cross‐over trials and non‐randomized intervention studies. To be included in this analysis, studies had to report at least one of the following at 1 week or longer from study start, and data had to be available based on observed or self‐reported use of EC and CC:

- Carbon monoxide (CO); we anticipated that this would be the most common measure among studies as it can be collected relatively easily
- Any other known biomarkers of potential harm (see Table 1)

**TABLE 1:** Effect direction plot for biomarkers of harm, by comparison group.

Biomarker class	Biomarker	Group comparisons		
		EC versus CC	EC versus EC + CC	Dual use (EC + CC) versus CC
Mercapturic acids	3‐HPMA (3‐hydroxypropylmercapturic acid)	↓↓ Cravo ↓↓ Morris	↔ McRobbie ↓ Goniewicz ↓↓ Morris ↔ Pulvers	↓↓ Morris
	SPMA (*S*‐phenylmercapturic acid)	↓↓ Cravo ↓↓ Morris	↓ Goniewicz ↓↓ Morris	↓↓ Morris
	HEMA (2‐hydroxyethylmercapturic acid)	↓↓ Morris	↑ Goniewicz ↔ Morris ↔ Pulvers	↓↓ Morris
	MHBMA (2‐hydroxy‐3‐buten‐1‐ylmercapturic acid)	↓↓ Morris	↓ Goniewicz ↓ Morris	↓↓ Morris
	HPMMA (3‐hydroxy‐1‐methyl propylmercapturic acid)		↓ Goniewicz ↔ Pulvers	
	AAMA (*N*‐acetyl‐*S*‐(carbamoylethyl)‐l‐cysteine (synonym: 2‐carbamoylethylmercapturic acid))		↓ Goniewicz ↓↓ Pulvers	
	CNEMA (2‐cyanoethylmercapturic acid)	↓↓ Morris	↓ Goniewicz ↓↓ Pulvers ↓↓ Morris	↓↓ Morris
	2‐HPMA (2‐hydroxypropylmercapturic acid)		↓ Goniewicz ↔ Pulvers	
	3‐HMPMA (3‐hydroxy‐1‐methylpropyl‐mercapturic acid)	↓↓ Morris	↓↓ Morris%60	↓↓ Morris
	PMA (phenylmercapturic acid)		↓↓ Pulvers	
	MMA (*N*‐nitrosodimethyamine)		↔ Pulvers	
Nitrosamines	NNAL (4‐(methylnitrosamino)‐1‐(3‐pyridyl)‐1‐butanol)	↓↓ Cravo ↓↓ Morris	↓ Goniewicz ↔ Morris ↓↓ Pulvers	↓↓ Morris
Metabolites of polyaromatic hydrocarbons	1‐Hydroxyfluorene		↑ Goniewicz	
	3‐, 4‐Hydroxyphenanthrenes		↑ Goniewicz	
	2‐Hydroxyfluorene		↑ Goniewicz	
	1‐Hydroxypyrene (1‐OHP)	↓↓ Morris	↑ Goniewicz ↓↓ Morris	↓↓ Morris
	3‐Hydroxyfluorene		↓ Goniewicz	
	2‐Hydroxyphenanthrene		↑ Goniewicz	
	1‐Hydroxyphenanthrene		↑ Goniewicz	
	2‐Naphtol		↓ Goniewicz	
Other known carcinogens	*o*‐tol (*o*‐toluidine)	↓↓ Morris	↓↓ Morris	↓↓ Morris
	1‐AN (1‐aminonaphthalene)	↓↓ Morris	↓↓ Morris	↓↓ Morris
	2‐AN (2‐aminonaphthalene)	↓↓ Morris	↓↓ Morris	↓↓ Morris
	NNN (*N*‐nitrosonornicotine)	↔ Morris	↔ Morris	↔ Morris
	3‐OH B[*a*]P (3‐hydroxybenxo[*a*]pyrene)	↓↓ Morris	↓↓ Morris	↓↓ Morris

Cravo et al. [8] reports data on a subset of participants who were confined for the first week of the study; all complied with study protocols during this period. Data are available in figures only; error bars do not overlap. In Gonieciwz et al. [9], all participants were given an electronic cigarette (EC) at baseline. After 2 weeks, statistically significant declines in 12 of 17 measured biomarkers of exposure to toxicants were observed. Authors also conducted a secondary analysis comparing changes at 2 weeks between those who switched completely to EC and those who used both EC and combustible cigarettes (CC). They give only absolute values (in their Supporting information, Table S3). In Morris et al. [6], all participants exclusively used EC at days 1–9 and then were randomized to three groups from days 10 to 14 (EC, CC and EC + CC); participants were confined for the duration of the study. Authors report between‐group differences and breakdown into two separate samples (studies 1 and 2). Study 1 has the larger sample size so is included in Table 1. Study 2 found consistent directions of effect for all markers. Pulvers et al. [13] assigned all participants to EC and reports median and interquartile ranges (IQRs) for a number of toxic exposures across three groups at 4 weeks: people who switched to EC for at least the first 2 weeks (*n* = 10); people who switched exclusively for the full 4 weeks (*n* = 6); and people who used both EC and CC throughout the entire 4‐week period (*n* = 21). The latter two groups are compared in Table 1. ↓↓ lower point estimate in EC group, with 95% confidence interval (CI)/error bars non‐overlapping; ↓ lower point estimate in EC group, with 95% CI/error bars/*P*‐value not provided; ↔ CI/error bars overlap; ↑ higher point estimate in EC group, with 95% CI/error bars/*P*‐value not provided)

Where outcome data were available at multiple time points, we used longest follow‐up.

### Screening, data extraction and risk of bias assessments

Two reviewers screened titles and abstracts, and then full texts, using the above criteria. Discrepancies were resolved by discussion or via referral to a third reviewer. We extracted study characteristics and carried out risk‐of‐bias assessments as part of the parent review following Cochrane methods (independently and in duplicate). One reviewer extracted outcome data for this analysis and a second reviewer checked it. We could not formally test for publication bias in this paper, as there were no meta‐analyses with 10 or more studies; the Cochrane review did not detect evidence of publication bias but could not rule it out.

### Analysis

Where data allowed, and more than one study reported on an outcome within a given comparison, we conducted inverse variance random‐effects meta‐analyses using RevMan version 5. Where both change from baseline and absolute values at follow‐up were available, we preferred change from baseline. Where studies presented absolute values at follow‐up only, we used these data and subgrouped by absolute values versus change, as recommended by Cochrane. We assessed statistical heterogeneity using the *I* ^2^ statistic; where this was above 75%, we considered effect direction when deciding appropriateness of reporting pooled estimates. For outcomes and comparisons where heterogeneity or issues with study reporting precluded meta‐analyses, we synthesized results using effect direction plots, where possible. For all other instances, we reported results narratively.

## RESULTS

Of the 49 studies measuring at least one biomarker of exposure identified via our parent Cochrane review, seven provided data relevant to these analyses, together with two new studies identified following publication of the parent review [5, 6]. In total, we include nine studies (*n* = 1299) in our syntheses (see Table 2 for summary, see Cochrane review for full characteristics [4] and see the Supporting information for flow diagram).

**TABLE 2:** Characteristics of included studies.

Study ID	Device type^a^	Total *N* (baseline)	Groups reported	Outcomes available (time‐point)	Data type	Overall risk of bias judgement	Tobacco or EC industry funding? (Y/N)	Country
Cobb *et al*. 2021 [7]	Cartridge	520	Covariates include CC and EC use	eCO (3 months)	Absolute values at follow‐up	Low	N	USA
Cravo *et al*. 2016 [8]	Cig‐a‐like	419	EC and CC	Multiple biomarkers (1 week)	Change from baseline	High	Y	UK
Goniewicz *et al*. 2017 [9]	Cig‐a‐like	22	EC and dual use	Multiple biomarkers (1 and 2 weeks)	Change from baseline	High	N	Poland
Ikonomidis *et al*. 2018 [10]	Not clear	90	EC, CC and dual use	eCO (1 month)	Absolute values at follow‐up	High	N	Greece
Kerr 2020 [5]	Refillable	55	EC and abstainers	eCO (12 weeks)	Absolute values at follow‐up	High	N	UK
McRobbie *et al*. 2015 [11]	Cig‐a‐like	40	EC, CC and dual use	3‐HPMA (4 weeks)	Change from baseline	High	N	UK
Morris *et al*. 2022 [6]	Pod	79	EC, CC and dual use	eCO and multiple biomarkers (2 weeks)	Absolute values at follow‐up	High	Y	USA
Pacifici *et al*.2015 [12]	Refillable	34	EC, CC and dual use	eCO (1, 4, 8 months)	Absolute values at follow‐up	High	N	Italy
Pulvers *et al*. 2018 [13]	Refillable	40	EC and dual use	Multiple biomarkers (4 weeks)	Change from baseline	High	N	USA

^a^ Based on classification in Hartmann‐Boyce *et al*. [4]; more information on product characteristics in each study can be found in the Cochrane review. Y/N = yes/no; EC = electronic cigarette; CC = combustible cigarette; eCO = exhaled carbon monoxide; HPMA = hydroxypropylmercapturic acid.

### Carbon monoxide

**FIGURE 1:**
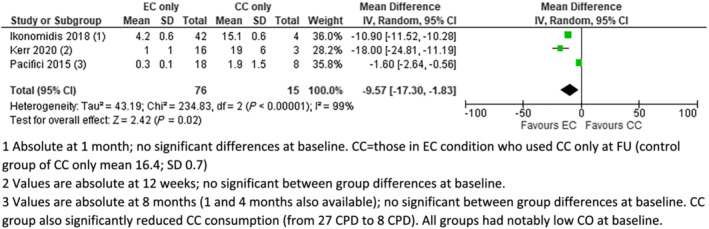
Exhaled carbon monoxide (eCO) [parts per million (p.p.m.)], electronic cigarettes (EC) versus combustible cigarettes (CC). (1) Absolute at 1 month; no significant differences at baseline. CC = those in EC condition who used CC only at follow‐up (FU) [control group of CC only mean 16.4; standard deviation (SD) = 0.7]. (2) Values are absolute at 12 weeks; no significant between‐group differences at baseline. (3) Values are absolute at 8 months (1 and 4 months also available); no significant between‐group differences at baseline. CC group also significantly reduced CC consumption [from 27 cigarettes per day (CPD) to eight CPD]. All groups had notably low CO at baseline

**FIGURE 2:**
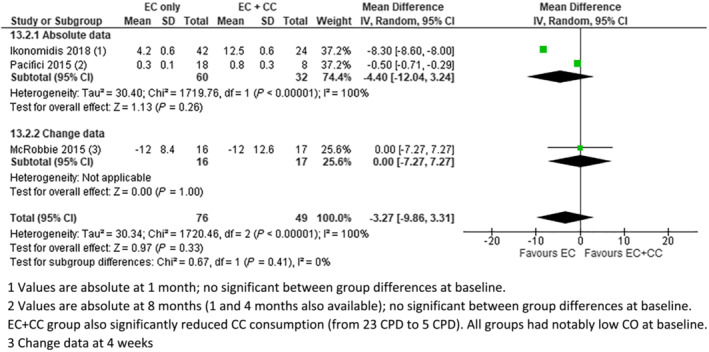
Exhaled carbon monoxide (eCO) [parts per million (p.p.m.)], electronic cigarettes (EC) versus EC + combustible cigarettes (CC). (1) Values are absolute at 1 month; no significant between‐group differences at baseline. (2) Values are absolute at 8 months (1 and 4 months also available); no significant between‐group differences at baseline. EC + CC group also had significantly reduced CC consumption [from 23 cigarettes per day (CPD) to five CPD]. All groups had notably low CO at baseline. (3) Change data at 4 weeks

**FIGURE 3:**
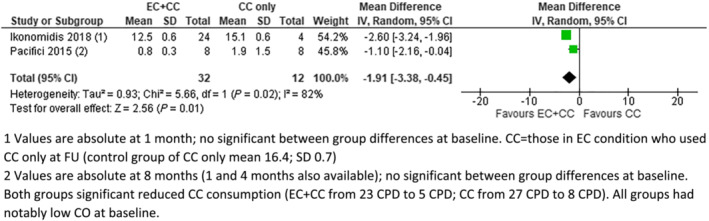
Exhaled carbon monoxide (eCO) [parts per million (p.p.m.)], electronic cigarettes (EC) + combustible cigarettes (CC) versus CC. (1) Values are absolute at 1 month; no significant between‐group differences at baseline. CC = those in EC condition who used CC only at follow‐up (FU) [control group of CC only mean 16.4; standard deviation (SD) = 0.7]. (2) Values are absolute at 8 months (1 and 4 months also available); no significant between‐group differences at baseline. Both groups significantly reduced CC consumption (EC + CC from 23 CPD to five CPD; CC from 27 CPD to eight CPD). All groups had notably low CO at baseline

Seven studies provided data on CO. Four of these provided data on eCO which could be meta‐analysed (Figs 1, 2, 3). In pooled analyses, eCO was lower in the EC group than in the dual use or CC groups, with 95% confidence intervals (CIs) excluding no difference in the comparison with CC. eCO was also statistically significantly lower when comparing dual use to CC only (Fig. 3). In all cases, *I* ^2^ indicated substantial statistical heterogeneity, but this was driven by magnitude rather than direction of effect, so we present pooled results.

Kerr 2020 was the only study to provide data on abstainers; eCO levels at 12 weeks were the same between the EC group (*n* = 16) and abstainers (*n* = 12), at 1 part per million (p.p.m.) [standard deviation (SD) = 1 in both groups] [5]. Abstainers had statistically significantly lower eCO at 12 weeks than the CC group (*n* = 3) [mean difference (MD) = −18.00 p.p.m., 95% CI = –24.81 to −11.19; analysis not shown).

Cobb et al. reported linear mixed effects models investigating product exposure variables [7]. They reported no statistically significant association between times ECs were used and eCO and a statistically significant positive association between eCO and cigarettes per day, at both 1 and 3 months (*P* < 0.02; data not reported). In Morris et al. [6], all participants exclusively used EC at days 1–9 and were subsequently randomized to three groups from days 10 to 14 (EC, CC and dual use); participants were confined for the duration of the study [6]. Authors reported between‐group differences in percentage saturation of carboxyhaemoglobin (COHb; a blood measure of CO): at day 14 (*n* = 14), levels were statistically significantly lower in EC compared to both CC and dual‐use [EC versus CC least squares (LS) MD = −7.13, 95% CI −8.41 to −5.85; EC versus dual‐use LS MD −2.56, 95% CI −3.81 to −1.31]. Levels were also statistically significantly lower in dual use compared to CC (LS MD −4.57, 95% CI −5.90 to −3.25).* Goniewicz et al. provided absolute values without measures of precision for eCO comparing EC to EC + CC at 2 weeks; both groups had reductions from baseline, with the reduction in the EC group reaching statistical significance (EC: 11 to 2 p.p.m., EC + CC 19 to 6 p.p.m.) [9].

### Other biomarkers

Five studies reported data on other biomarkers of potential harm; none reported data on abstainers. Only one presented data that could have been meta‐analysed; in McRobbie et al. [11], both EC and dual use groups showed statistically significant reductions in 3‐hydroxy‐1‐methylpropyl‐mercapturic acid (3‐HMPA) at 4 weeks, with no statistically significant between‐group difference in change from baseline (MD = 194.00 ng/g, 95% CI = −502 to 890) [11]. Results throughout studies are presented in an effect direction plot (Table 1). The majority of studies showed absolute reductions from baseline in both EC and EC + CC groups (see Cochrane review and individual papers for more detail); Table 1 reflects between‐group differences.

Of the 13 biomarkers measured when comparing EC versus CC, 12 were lower in EC groups; the one study measuring *N*‐nitrosonornicotine (NNN) did not detect a statistically significant difference (Table 1) [6]. Of the 25 measured when comparing EC to dual use, results were lower for EC groups for 12 measures (95% CI/error bars non‐overlapping) and dual use for five biomarkers (statistical significance unclear). For the remaining eight measures, single studies did not detect statistically significant differences between groups, or the multiple studies contributing to the outcome had inconsistent results. Only one study provided data comparing dual use to CC; of the 13 biomarkers measured, 12 were lower in EC, with no statistically significant difference detected for NNN [6].

## DISCUSSION

In these secondary data analyses, combining data from studies in which people who smoked were provided with ECs, exclusive EC use was associated with lower levels of biomarkers of harm than exclusive use of combustible tobacco or than dual use. This is consistent with other studies and broad scientific consensus that the greatest improvements in health come from ceasing combustible tobacco use in its entirety and that, although not completely without risk, EC are a reduced‐risk alternative [1, 2].

Concerns have been cited by academics and policymakers that using both EC and combustible tobacco may lead to more harm than exclusive combustible tobacco use [2]. Historically, this has been a barrier to research studies and policies that advocate provision of EC to people who smoke as a harm reduction strategy. However, the studies analysed in this review showed no evidence that biomarkers of potential harm increased in people who continued to smoke combustible tobacco while also using EC. In fact, dual use was associated with statistically significant reductions in CO, nitrosamines (mixed evidence), some metabolites of polyaromatic hydrocarbons and some mercapturic acids. However, for all outcomes but CO, data were very limited, and these limitations precluded meta‐analysis. For CO, we observed substantial statistical heterogeneity, but this was driven by the magnitude as opposed to the direction of effect. Concerns have been voiced around dual use leading to greater levels of nicotine exposure than using either e‐cigarettes or conventional cigarettes alone. We do not analyse nicotine exposure here (as it is not necessarily a measure of harm); separate research is needed on this topic.

By their very nature, the data included in these analyses either came from short‐term confinement studies, which are not readily generalizable to real‐world use, or from non‐randomized groupings, which introduce the possibility of unmeasured confounding. However, the findings presented here are consistent with those from the parent Cochrane review, which relied on randomization, and with evidence that if people who smoke obtain nicotine from alternative sources, they smoke less [14]. Evidence is also consistent with that from many observational studies, with reductions in biomarkers observed in EC users compared to people smoking combustible tobacco. A systematic review of nine studies examining the association between nicotine device type and biomarkers of harm found that levels of toxicants were lower in people using EC than in people using combustible cigarettes. The authors deemed this an indication of reduced levels of the following harmful chemicals: butadiene, acrolein, benzene, toluidine, naphthylamine and methylnitrosamines [15]. We encourage studies to collect information on other potential biomarkers that may more clearly characterize EC‐specific health risks that we may be missing. More studies are also needed to quantify how biomarkers differ in abstainers compared to EC groups; only Kerr [5] provided data on abstainers that we could use in our analyses.

A further limitation to our analysis is variation in the products used in these studies, including product types and use characteristics of individuals. Assessing the harmfulness of EC components is difficult because their design and product characteristics vary. Further analyses need to be undertaken examining associations between device type, flavouring and nicotine content on biomarker exposure.

Finally, our main limitation is that we were unable to access individual participant data on exposures. Such data are needed to further examine relationships between EC use patterns, including levels of dual use, and biomarkers of potential harm.

## DECLARATION OF INTERESTS

A.R.B., A.T., J.H.B., N.L., I.O.: none to declare. P.H. provided consultancy to and received research funding from Pfizer. C.B. is an investigator on grants and contracts for research on tobacco control from the Health Research Council of NZ, the NHMRC Australia, NZ Ministry of Health and Auckland Council. He has provided consultancy to J&J Japan on NRT products. N.A.R. has received royalties from UpToDate, Inc., for chapters on electronic cigarettes. Outside the topic of e‐cigarettes, she has consulted for and received a research grant from Achieve Life Sciences.

## AUTHOR CONTRIBUTIONS

**Jamie Hartmann‐Boyce:** Conceptualization; data curation; formal analysis; funding acquisition; investigation; methodology. **Ailsa R. Butler:** Conceptualization; data curation; funding acquisition. **Annika Theodoulou:** Data curation; investigation. **Peter Hajek:** Investigation; methodology. **Chris Bullen:** Investigation; methodology. **Nancy A. Rigotti:** Investigation; methodology. **Nicola Lindson:** Conceptualization; data curation; formal analysis; funding acquisition; investigation; methodology.

## Supporting information


**Data S1.** Supporting Information

## ENDNOTE

* Data reported separately for two samples: study 1 and study 2. These data are from study 1; study 2 data have a smaller sample but are all consistent with study 1 findings for COhB.
