# Supplementary material for: Biomarkers of potential harm in people switching from smoking tobacco to exclusive e‐cigarette use, dual use or abstinence: secondary analysis of Cochrane systematic review of trials of e‐cigarettes for smoking cessation
Source: Addiction. 2022 Oct 21;118(3):539–45. doi: 10.1111/add.16063 (PMC10092879; doi:10.1111/add.16063)
Supplement: Supplementary file 1 — Data S1. Supporting Information [file ADD-118-539-s001.docx]

**Biomarkers of potential harm in people switching from smoking tobacco to exclusive e-cigarette use, dual use, or abstinence: Secondary analysis of Cochrane systematic review of trials of e-cigarettes for smoking cessation**

**Supplementary Figure 1: Flow diagram of studies with data on at least one biomarker of exposure.**

**Cochrane Review Sept 2021 July to January 2022 search findings**

61 completed studies included in Cochrane Review published 2021

12 studies not relevant

821 records imported for screening. July to Nov 2021 searches

86 duplicates removed

689 records irrelevant

735 records screened

46 full-text articles assessed for eligibility

31 articles excluded

42 studies not relevant to these analyses

49 studies included with data on at least one biomarker of exposure

15 articles included as eligible for the Cochrane Living Systematic Review (new study (4), linked study (7), ongoing study (4))

13 records not relevant

7 study included with data on at least one biomarker of exposure relevant to these analyses

2 studies included with data on at least one biomarker of exposure

9 studies included with data on at least one biomarker of exposure relevant to these analyses
